# Wavelength-Switchable Ytterbium-Doped Mode-Locked Fiber Laser Based on a Vernier Effect Filter

**DOI:** 10.3390/mi15111289

**Published:** 2024-10-23

**Authors:** Hailong Xu, Liqiang Zhang, Xiangdong Li, Jiaxin Li, Yuanzhen Liu, Yicun Yao, Minghong Wang

**Affiliations:** School of Physics Science and Information Technology, Liaocheng University, Liaocheng 252000, China; 2220110605@stu.lcu.edu.cn (H.X.); 2310110404@stu.lcu.edu.cn (X.L.); 2320110604@stu.lcu.edu.cn (J.L.); 2320110606@stu.lcu.edu.cn (Y.L.); yaoyicun@lcu.edu.cn (Y.Y.)

**Keywords:** passively mode-locked fiber laser, Vernier effect, nonlinear polarization rotation, Mach–Zehnder interferometer, wavelength switchable

## Abstract

A wavelength-switchable ytterbium-doped mode-locked fiber laser is reported in this article. Two Mach–Zehnder interferometers (MZIs, denoted as MZI1, MZI2) with close free spectral ranges (FSRs) are connected in series to form a Vernier effect sensor. By utilizing the filtering effect of the Vernier effect sensor, the wavelength-switchable output of an ytterbium-doped mode-locked fiber laser is realized. When the 3 dB bandwidth of the Vernier effect filter is set to be 5.31 nm around 1073.42 nm, stable dissipative solitons are obtained. Stretching MZI1 horizontally, the central wavelengths of the pulses can be switched among 1073.42 nm, 1055.38 nm, and 1036.22 nm, with a total tunable central wavelength range of 37.2 nm. When the 3 dB bandwidth of the Vernier effect filter is set to be 4.07 nm, stable amplifier similaritons are obtained. Stretching MZI1 horizontally, the central wavelengths of the pulses are switchable among 1072.71 nm, 1060.15 nm, 1048.92 nm, and 1037.26 nm, with a total tunable central wavelength range of 35.15 nm. Compared with traditional fiber interference filters, the Vernier effect filter has a higher sensitivity, making wavelength switching more convenient and providing a wider tuning range for the ytterbium-doped mode-locked fiber laser.

## 1. Introduction

Mode-locked fiber lasers produce high-intensity, ultra-short pulse lasers with remarkable single-pulse energy. These lasers have found extensive applications in various fields, including precision machining, biomedicine, and the forefront of scientific research [1,2]. According to the gain medium, mode-locked fiber lasers operate at different wavelengths. Erbium-doped fiber lasers operate near 1.5 μm [3,4,5,6] and are widely used in the fields of fiber communications, fiber sensing, and optical frequency metrology. Thulium-doped fiber lasers operate near 2.0 μm [7] and are mainly used for operations within the safe wavelength range of the human eye. Ytterbium-doped mode-locked fiber lasers operating around 1.0 μm [8,9,10], with a wide gain range and narrow pulse duration, have a wide range of applications in the fields of precision machining, ultrafast optics and so on. Compared with single-wavelength mode-locked fiber lasers, wavelength-tunable or -switchable lasers offer a distinct advantage by providing laser pulses at various central wavelengths. These lasers are not only cost-effective but also user-friendly, making them an attractive choice for a wide range of applications. To achieve tunable or switchable laser wavelengths, wavelength selection devices are typically incorporated into the laser system to counteract the effects of gain competition within the gain medium. Diffraction gratings are commonly used as beam-splitting devices, which enable wavelength tuning in ytterbium-doped mode-locked lasers [11,12]. However, the incorporation of this discrete device does introduce a degree of complexity to the laser system. Long-period fiber gratings represent an all-fiber solution that seamlessly integrates with other fiber-based devices, as referenced in [13,14]. However, the fabrication of fiber gratings requires expensive and complex inscription equipment. Using the birefringence-induced filtering effect in the laser cavity [15], wavelength switching or tuning have also been achieved in fiber lasers, but the birefringence of the fiber is susceptible to environmental influences.

Another class of widely utilized filtering devices is the fiber interference filters, with the Mach–Zehnder interferometer (MZI) filter being particularly notable for its straightforward structure and ease of implementation [16,17,18,19]. In 2021, a switchable multichannel ytterbium-doped mode-locked fiber laser was demonstrated based on a MZI filter. The MZI filter was fabricated by splicing a small section of nonlinear-photonic-crystal fiber between two segments of single-mode fibers [16]. In 2024, by wrapping a section of single-mode fiber into two elliptical loops, a figure-eight knot-based MZI was established. Inserting the MZI filter into a mode-locked fiber laser, switchable and adjustable-spacing multiwavelength nanosecond pulses have been obtained [17]. In 2023, we fabricated two kinds of MZI filters with a tapered seven-core fiber (SCF) [18] and cascaded 3 dB couplers [19], and stable dissipative solitons and amplifier similaritons were obtained in ytterbium-doped mode-locked lasers. All of these lasers are tunable by applying tensile strain to the filters. Interference filters achieve wavelength tuning through the change of the ambient physical quantities, such as stretching or bending. If the interferometer has a higher sensitivity, wavelength tuning will be more convenient, and the tuning range will be wider.

Recently, the Vernier effect has been widely used in the field of fiber sensing. Typically, a sensing system with a Vernier effect consists of two interferometers with similar free spectral ranges (FSRs) [20,21]. In general, one of the interferometers acts as the reference interferometer and the other is the sensing interferometer. The transmission curves of the two interferometers are superimposed to form a periodic envelope. A small shift in the transmission curve of the sensing interferometer leads to a significant shift in the superimposed envelope, resulting in an exponential increase in sensing sensitivity. Various fiber sensors based on the Vernier effect have been developed using MZIs [22,23], Sagnac interferometers [24], Michelson interferometers [25], and Fabry–Perot interferometers [26], which have significantly improved the sensitivity of fiber sensors. The use of highly sensitive interferometers as filters will make the tuning/switching of laser wavelengths easier.

In this paper, in order to make it easier for the filter to achieve wavelength tuning and switching in fiber lasers, Vernier effect filters were prepared and inserted into ytterbium-doped mode-locked lasers for the first time. That is, two conical SCF MZIs, denoted as MZI1 and MZI2, were connected in series to form a Vernier effect sensor, which was used as a filter in ytterbium-doped mode-locked fiber lasers. When the FSRs of MZI1 and MZI2 are 17.2 nm and 15.0 nm, respectively, stable three-wavelength switchable dissipative solitons can be obtained, with a total central wavelength-switching range of 37.2 nm. When the FSRs of MZI1 and MZI2 are 12.6 nm and 10.0 nm, respectively, stable four-wavelength switchable amplified self-similar pulses can be obtained, with a total and central wavelength switching range of 35.15 nm. Compared with a single MZI filter, mode-locked lasers based on the Vernier effect can achieve a wider wavelength switching range with a smaller stretching amount, making wavelength switching more convenient.

## 2. Principle of Vernier Effect

Mechanical Vernier calipers, which include fixed and sliding scales, can enhance the precision of measurements. The optical Vernier effect works similarly to mechanical Vernier calipers by combining two interferometers with approximately the same FSRs. One of the interferometers acts as the sensing part, corresponding to the sliding scale, and the other interferometer acts as the reference part, corresponding to the fixed scale. When the interference spectra of these two interferometers are superimposed on each other, their peaks and dips interfere with each other to form a new envelope spectrum [27]. The FSR of the envelope can be expressed as a function of the FSRs of the two interferometers with the following equation:

(1) $$FSR_{envelope}=\left|\frac{FSR_{s}FSR_{r}}{FSR_{s}-FSR_{r}}\right|$$

where $FSR_{r}$ and $FSR_{s}$ are the FSRs of the reference and sensing interferometers, respectively. The sensitivity of sensing can be significantly improved by tracking the amount of drift of the envelope peak with the external environment. The amplification factor of the sensitivity can be roughly calculated by the following equation.

(2) $$M=\left|\frac{FSR_{r}}{FSR_{s}-FSR_{r}}\right|=\frac{L_{s}}{\left|L_{r}-L_{s}\right|}$$

where $L_{r}$ and $L_{s}$ are the optical path difference of the reference interferometer and sensing interferometer, respectively.

**Figure 1:**
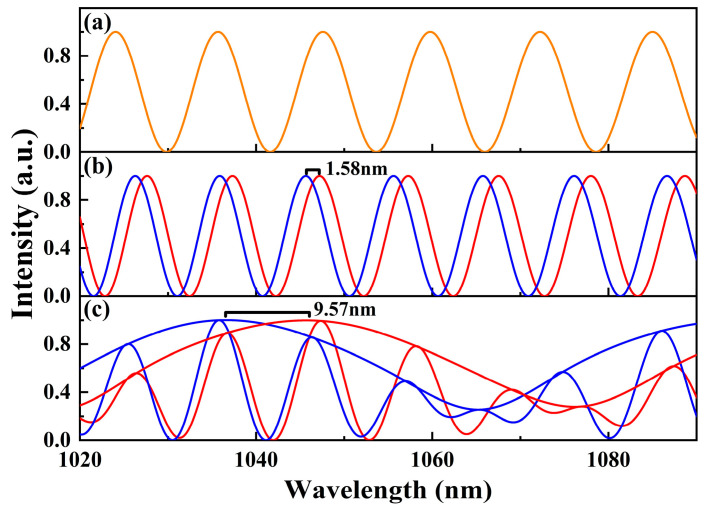
Calculated transmission cures of MZI1 (**a**) and MZI2 (**b**), and the superimposed spectra (**c**). The blue curves in (**b**,**c**) are the results of changing the optical path length of MZI2.

In order to understand the Vernier effect more deeply, we carried out numerical simulations. The transmission curves of these two MZIs and the superimposed spectra are shown in Figure 1. MZI1 is the reference interferometer with the orange curve in Figure 1a and MZI2 is the sensing interferometer with the red curve in Figure 1b. The superimposed spectra with the obvious envelops are shown in Figure 1c. When the optical path difference of MZI2 is changed, the interference curve is blue-shifted by 1.58 nm. The envelope of the superimposed spectrum is blue-shifted by 9.57 nm, and the sensitivity is amplified by a factor of 6.1, which agrees with the calculated result according to Equation (2). It can be seen that the optical Vernier effect can significantly improve the sensitivity of the sensor. Applying a Vernier effect sensor as a filter in a mode-locked fiber laser, the wavelength switching will be more convenient and the switching range will be wider.

## 3. Experimental Setup

**Figure 2:**
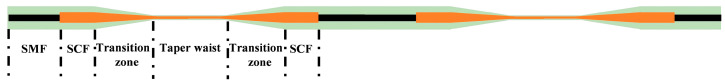
Schematic diagram of the Vernier effect filter based on tapered SCF MZIs.

In the experiment, a MZI filter is prepared by fusing a section of tapered SCF between two sections of single-mode fibers [18]. To fabricate the SCF MZI, a 20 cm long SMF is first fused to a section of SCF with a fiber-optic fusion splicer. Then, the SCF is stripped of all the coating layer and retained for about 1.5 cm to be fused to another 20 cm long SMF. Next, the two ends of the SMF are fixed on the displacement table of the cone pulling machine by clamps. The middle SCF is heated with a hydroxide flame, and the two displacement tables are used to stretch the fiber in opposite directions. During the cone pulling process, we use a spectrometer to monitor the transmission spectrum. The heating and stretching are stopped when the desired FSR is reached, and the UV adhesive is subsequently utilized to secure the tapered SCF to the U-frame. Figure 2 shows the schematic diagram of the Vernier effect filter based on tapered SCF MZIs. The SCF is originally designed to enlarge the transmission capacity by spacing division multiplexing, and each of the seven cores transmits signals along the fiber independently. When a part of the SCF is tapered, the core diameter and the spacing between adjacent cores are reduced, and this tapered SCF is turned into a strongly coupled multi-core fiber. Supermodes are then excited to generate interference in the tapered SCF.

To analyze the operating principle of the supermode interference in the tapered SCF, a coupled mode equation is employed. When the light is launched into the central core of the tapered SCF, the coupled equation can be written as [28]

(3) $$\frac{d\vec{A}}{dz}=-\vec{C}\vec{A}\left(z\right)$$

where $\vec{A}={\left[A_{1}\left(z\right)A_{2}\left(z\right)A_{3}\left(z\right)A_{4}\left(z\right)A_{5}\left(z\right)A_{6}\left(z\right)A_{7}\left(z\right)\right]}^{T}$ is the column vector of the amplitude of each core mode of the tapered SCF. $\vec{C}$ is the coupling coefficient. Assuming that the coupling between the cores is negligible due to the large inter-core distance, the intensities of the normalized center core mode and the side-core modes can be derived if light is emitted into the central core [28]:

(4) $${\left|A_{1}\left(z\right)\right|}^{2}=\frac{1}{7}+\frac{6}{7}\cos^{2}\left(\sqrt{7}Cz\right)$$

(5) $${\left|A_{p}\left(z\right)\right|}^{2}=\frac{1}{7}\sin^{2}\left(\sqrt{7}Cz\right)\;p\neq 1$$

**Figure 3:**
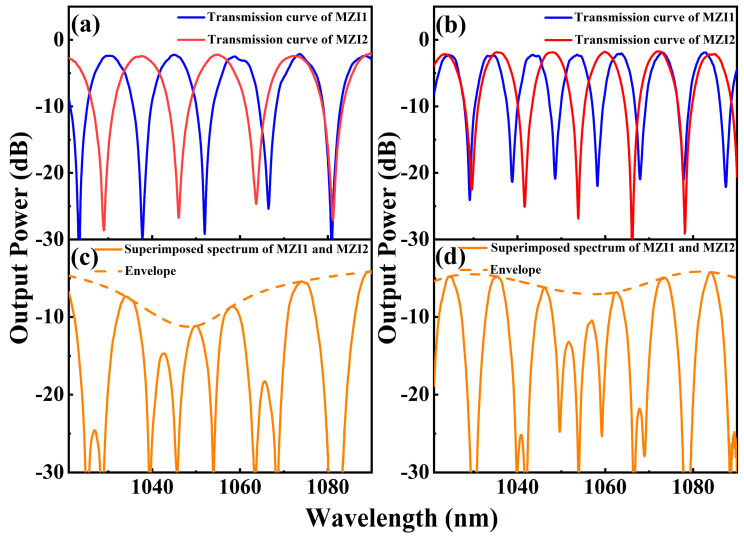
(**a**,**b**) Transmission curves of MZI1 and MZI2 with different FSRs. (**c**,**d**) Superimposed spectra of MZI1 and MZI2.

The coupling coefficient *C* can be derived as follows [28]:

(6) $$C=\frac{\pi }{2}\frac{\sqrt{n_{1}^{2}-n_{2}^{2}}}{an_{1}}\frac{U^{2}}{V^{2}}\frac{K_{0}\left(Wd/a\right)}{K_{1}^{2}\left(W\right)}$$

where $n_{1}$ and $n_{2}$ are the refractive indices of the core and cladding modes, respectively. a and d are the core diameter and the distance between two cores, respectively. $K_{0}$ and $K_{1}$ represent the zero- and first-order Henkel functions. U, W and V are, respectively, the normalized radial phase constant, the normalized radial attenuation constant, and the normalized frequency. From Equations (4) and (5), it is evident that the central core mode and the side-core modes can be beat periodically with a phase difference of π/2. The variation of the tapered fiber diameter changes the core diameter and the spacing between two cores, resulting in the variation of the coupling strength among multiple cores. The FSR of the interference curve can be expressed as follows:

(7) $$FSR=\frac{\lambda^{2}}{\Delta_{\mathrm{neff}}\cdot \Delta L}$$

where λ is the transmission wavelength, $\Delta_{\mathrm{neff}}$ is the effective refractive index difference between different modes of the light waves, and ΔL is the optical path difference between different modes. The effective refractive index difference increases as the diameter of the tapered region decreases, resulting in a decrease in FSR. Therefore, the FSR of the filter can be controlled according to the diameter of the tapered region during the pulling process. Two Vernier effect filters with different FSRs are prepared in the experiment. The FSRs of MZI2 and MZI1 of the first Vernier effect filter are 15.0 nm and 17.2 nm, respectively. Transmission curves of these two MZIs and the superimposed spectrum are shown in Figure 3a,c. As shown in Figure 3c, there is an obvious envelope appearing in the superimposed spectrum. The 3 dB bandwidth around 1073.42 nm is measured to be 5.31 nm. The FSRs of MZI2 and MZI1 of the second Vernier effect filter are 10.0 nm and 12.6 nm, respectively. The corresponding transmission curves are shown in Figure 3b,d. The 3 dB bandwidth of the cascaded transmission curve near 1072.72 nm is 4.07 nm. Stretching MZI1 in the experiment, a small drift of the transmission curve of MZI1 causes a significant drift of the envelope of the superimposed spectrum, making wavelength tuning more convenient.

**Figure 4:**
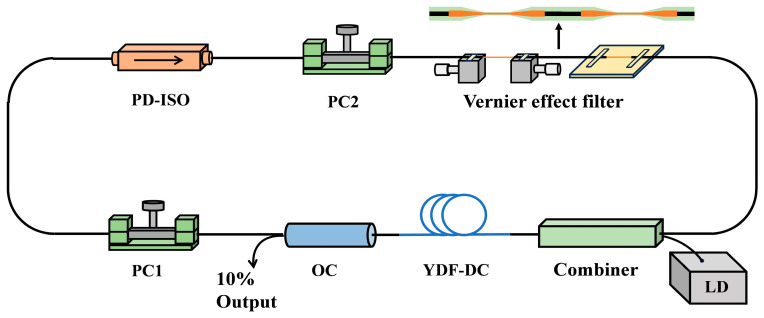
Schematic diagram of the mode-locked fiber laser based on the Vernier effect filter.

Figure 4 shows the experimental setup of the proposed mode-locked fiber laser. The fiber laser consists of a semiconductor laser, a pump/signal combiner, a section of 2.2 m Yb-doped double-cladding gain fiber (YDF-DC), a 10/90 fiber optical coupler (OC), two squeezed polarization controllers (PC1, PC2), a polarization-dependent isolator (PD-ISO), and the MZI-based Vernier effect filter. The pump light from the semiconductor laser is coupled into the gain fiber through the pump/signal combiner. The core and cladding diameters of the gain fiber are 10 μm and 125 μm, respectively, and the absorption coefficient at 976 nm is 7.4 dB/m. A coupler with a splitting ratio of 10/90 is used in the laser to output 10% of the light outside the cavity for signal measurements. Two squeezed polarization controllers and the polarization-dependent isolator are used to achieve nonlinear polarization rotational mode-locking, and the isolator also ensures unidirectional laser transmission. The Vernier effect filter enables pulse shaping in the frequency domain. The tapered SCF MZI with a small FSR is fixed by UV adhesive onto two one-dimensional displacement stages, with a precision of 10 μm. The optical path difference between different modes transmitted in the tapered SCF is changed during the stretching process, which results in the change of the wavelength peak of the transmission curve.

During the experiment, the spectra of the pulse are recorded by an optical spectrum analyzer (YOKOGAWA, AQ6374, Tokyo, Japan). An autocorrelator (FEMTOCHROME, FR-103XL, Berkeley, CA, USA) is utilized to measure the pulse duration. A 200 MHz digital storage oscilloscope (GWINSTEK, GDS-2202E, Taiwan, China) and an InGaAs PIN photodetector (NEWPORT, 818-BB-35, CA, USA) are used to monitor the pulse trains. The stability of the laser operation is evaluated by an RF spectra analyzer (KEYSIGHT, N9000B CXA, Santa Clara, CA, USA). Finally, the output power of the laser is measured by an optical power meter.

## 4. Experimental Results and Discussion

**Figure 5:**
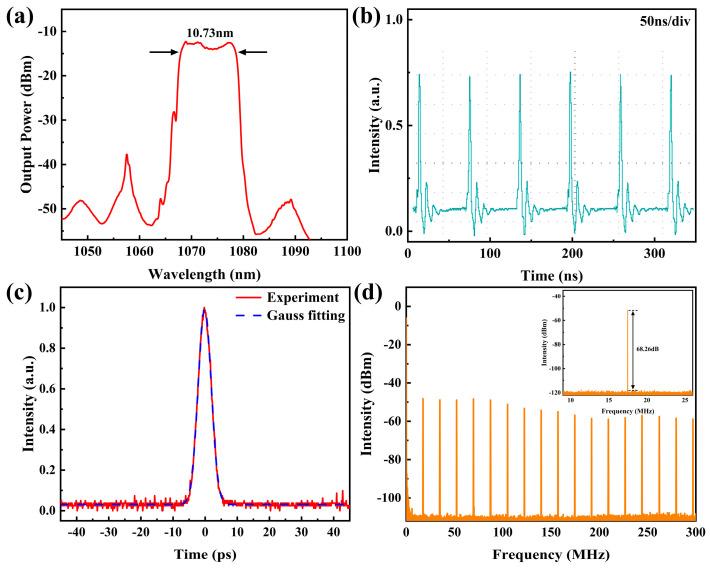
Characteristics of the dissipative solitons. (**a**) Spectrum; (**b**) Pulse train; (**c**) Autocorrelation trace; (**d**) RF spectra.

In the experiment, the first Vernier effect filter with FSRs of 15.0 nm and 17.2 nm is first inserted into the fiber laser. When the pump power is increased to 1.5 W, a stable mode-locked pulse is obtained by rotating the two polarization controllers. Figure 5 shows the characteristics of the pulses measured at a pump power of 1.6 W. Figure 5a shows the output spectrum of the pulse, which has a central wavelength of 1073.42 nm and a 3 dB bandwidth of 10.73 nm. The spectrum shows steep edges on both sides, which is an obvious feature of dissipative solitons. Figure 5b shows the mode-locked pulse train measured by the oscilloscope, with a repetition frequency of 17.46 MHz. Figure 5c shows the autocorrelation trace of the pulses measured by the autocorrelator, and the pulse duration is estimated to be 4.8 ps. Figure 5d shows the RF spectra of the pulses with a signal-to-noise ratio of 68.26 dB, indicating that the laser is operating in a stable mode-locked state. The scanned range is from 0 to 300 MHz, with a resolution bandwidth of 100 Hz. In the inset, the scanned range is from 9 to 26 MHz, with a resolution bandwidth of 10 Hz. The average output power of the laser measured by the power meter is 76 mW, corresponding to a single-pulse energy of 4.35 nJ.

**Figure 6:**
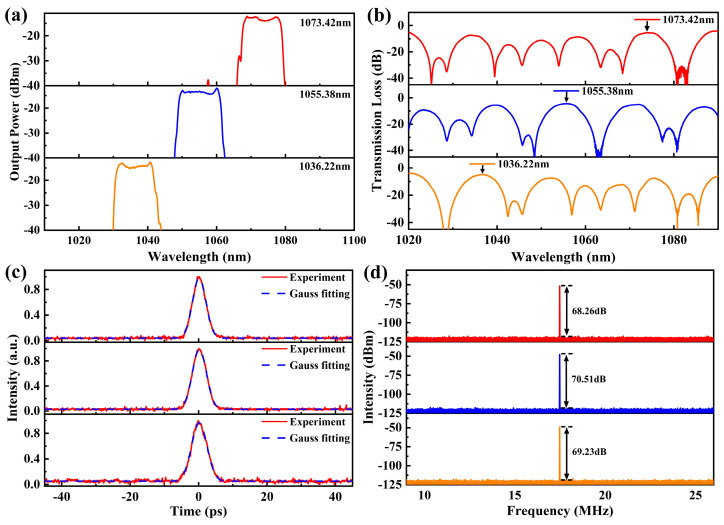
Characteristics of the three-wavelength switchable pulses. (**a**) Spectra; (**b**) Transmission curves of the Vernier effect filter; (**c**) Autocorrelation traces; (**d**) RF spectra. The red, blue, and orange curves in (**a**), (**b**), and (**d**) respectively correspond to the spectra, the transmission curve of the filter, and the RF spectra of different wavelengths.

**Figure 7:**
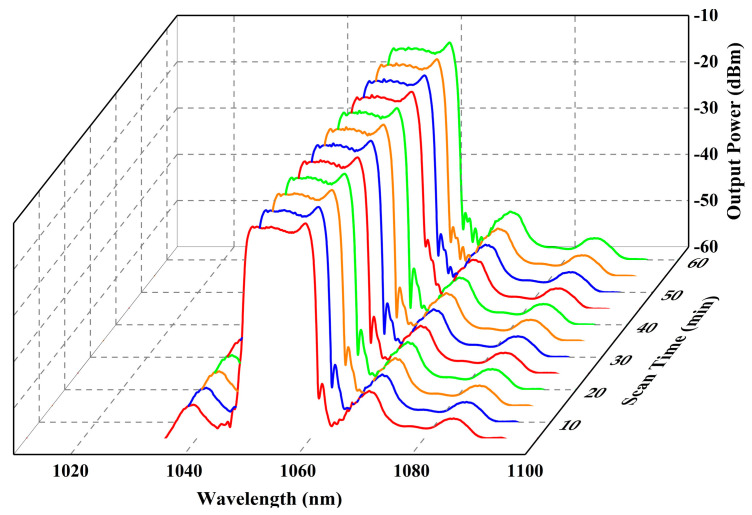
Spectra scanned repeatedly within one hour when the stretching amount is 0.04 mm.

Then, the pump power is kept constant at 1.6 W. Applying a tensile strain to the two ends of MZI1, the central wavelength of the mode-locked fiber laser can be switched between three wavelengths. Figure 6a shows the spectra with central wavelengths of 1073.42 nm, 1055.38 nm, and 1036.22 nm, respectively. The corresponding 3 dB bandwidths are 10.73 nm, 11.68 nm, and 10.73 nm, respectively, with a total tunable wavelength range of 37.2 nm. The total stretching amount of the fiber is 0.08 mm. Figure 6b shows the transmission curves of the Vernier effect filter when the stretching amount are 0, 0.04 mm and 0.08 mm, respectively, and the peak wavelengths of the transmission curves are in agreement with the central wavelengths of the spectra shown in Figure 6a. The autocorrelation traces of the pulses are shown in Figure 6c, and the pulse durations are 4.80 ps, 5.01 ps, and 5.13 ps, respectively. Figure 6d shows the RF spectra of the pulse train, and the signal-to-noise ratios are 68.26 dB, 70.51 dB, and 69.23 dB, respectively, which indicates that the laser is working in a stable mode-locked state. To investigate the stability of the mode-locked fiber laser, the monitoring of the central wavelength drift is conducted over a period of 1 h. The stretching amount is kept constant at 0.04 mm. The spectral information is recorded every 5 min for 1 h at room temperature. The results are shown in Figure 7. As shown in Figure 7, the maximum shift of the central wavelength in 1 h is less than 0.2 nm.

**Figure 8:**
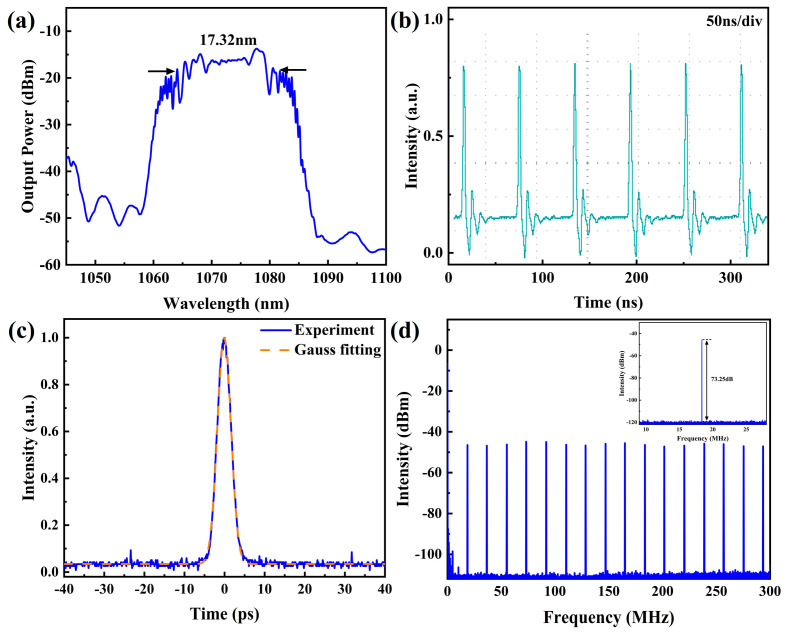
Characteristics of the amplifier similaritons. (**a**) Spectrum; (**b**) Pulse train; (**c**) Autocorrelation trace; (**d**) RF spectra.

Then, the prepared second Vernier effect filter with FSRs of 10.0 nm and 12.6 nm is inserted into the fiber laser. When the pump power is set to be 1.6 W, stable mode-locked pulses are obtained by adjusting the polarization controllers. The pulse characteristics are shown in Figure 8. As shown in Figure 8a, the central wavelength locates at 1072.71 nm, and the corresponding 3 dB bandwidth is 17.32 nm. Different from Figure 5a, the spectrum shows obvious modulation structures. The edges of the spectrum are no longer steep, and the overall shape of the spectrum is closer to a parabolic shape. Compared with the spectra reported in Ref. [18], we classify this type of pulses as amplifier similaritons. Figure 8b shows the pulse train recorded by the oscilloscope, and the repetition frequency is measured to be 18.35 MHz, corresponding to a cavity length of 11.35 m. The autocorrelation trace of the pulse is shown in Figure 8c, and the pulse duration is evaluated to be 4.27 ps. Figure 8d shows the RF spectra of the pulse, with the same scanning range and resolution bandwidth shown in Figure 5d. The signal-to-noise ratio of the RF spectra is about 73.25 dB, which indicates a stable mode-locked state of the fiber laser. The average output power is measured to be 80 mW, and the corresponding pulse energy is 4.36 nJ.

**Figure 9:**
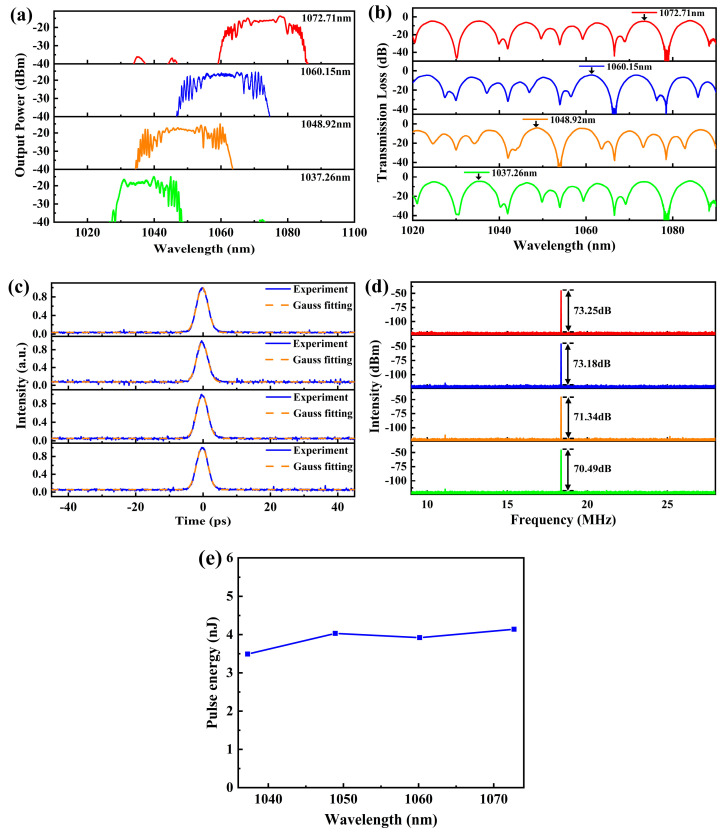
Characteristics of the four-wavelength switchable pulses. (**a**) Spectra; (**b**) Transmission curves of the Vernier effect filter; (**c**) Autocorrelation traces; (**d**) RF spectra. (**e**) Dependence of pulse energy on central wavelength. The red, blue, orange, and green curves in (**a**,**b**,**d**) correspond to the spectra, the transmission curve of the filter, and the RF spectra of different wavelengths.

By applying a tensile strain on MZI1, the central wavelength of the fiber laser can be switched between 1072.71 nm, 1060.15 nm, 1048.92 nm, and 1037.26 nm. The results are shown in Figure 9a. The total stretching amount is 0.11 mm, and the tunable range of the central wavelengths is 35.15 nm. The corresponding 3 dB bandwidths are 17.32 nm, 22.02 nm, 23.24 nm, and 16.41 nm, respectively. Figure 9b shows the transmission curves of the Vernier effect filter when the stretching amounts are 0.04 mm, 0.06 mm, 0.08 mm, and 0.11 mm, respectively. And again, the central wavelength of the fiber laser is in agreement with the peak wavelengths of the transmission curves. Figure 9c shows the measured autocorrelation traces, and the pulse duration varies from 4.27 ps to 5.40 ps. Figure 9d shows the RF spectra at different wavelengths, and the signal-to-noise ratios are all above 70 dB, indicating that the laser is continuously operating in a stable mode-locked state. Figure 9e shows the relationship between the pulse energy and central wavelength.

During the experimental process, we repeated the experiment several times and restored the tapered SCF to its initial position after each stretching. After each restoration, the polarization controller was adjusted to bring the laser back to the mode-locked state, and the central wavelength would be restored to its initial wavelength.

Experimentally, MZI2, which has the smaller FSR, is stretched. As the degree of stretching increases, the FSR of MZI2 diminishes, consequently reducing the FSR of the envelope as per Equation (1). Similarly, if MZI1, with its larger FSR, is stretched, its FSR also decreases with greater stretch, leading to an increase in the FSR of the envelope as per Equation (1). Stretching either MZI1 or MZI2 can induce envelope drift and facilitate wavelength switching.

We have compared the stretch amount and tuning range of the fiber laser presented in this paper with those reported in various studies utilizing different interference filters, as detailed in Table 1. In reference [18], an ytterbium-doped mode-locked fiber laser with an SMF-SCF-SMF filter configuration was employed, which exhibited a total tunable spectral range of 12.36 nm and a stretch amount of 0.29 mm. Reference [29] describes an ytterbium-doped mode-locked fiber laser featuring an SMF and multi-mode fiber split structure, with a total tunable wavelength range of 12.00 nm and a stretch amount of 0.504 mm. In contrast to the configurations in references [18,29], our developed filter achieves multi-wavelength switching with a minimal stretch of 0.08 mm. Furthermore, reference [30] reports on an erbium-doped mode-locked fiber laser incorporating an integrated tapered fiber wavelength tuner. Although the stretch applied to the filter is comparable to ours, its total tunable spectral range is limited to 4.40 nm, whereas our filter offers a significantly broader tunable range of 37.2 nm. This underscores the effectiveness of incorporating a Vernier effect filter into a mode-locked fiber laser for achieving a broader wavelength switching range with a minimal amount of stretch. The wavelength switching process is more facile, and the range of switching is substantially wider.

## 5. Conclusions

In summary, we demonstrate a multi-wavelength-switchable ytterbium-doped mode-locked fiber laser that incorporates a tapered SCF MZI-based Vernier effect filter. Stable dissipative solitons and amplifier similaritons have been obtained by inserting two Vernier effect filters with different FSRs into the fiber laser. Stretching one of the MZIs of the Vernier effect filter, the mode-locked dissipative solitons can be three-wavelength switched between 1073.42 nm, 1055.38 nm, and 1036.22 nm, with a total central wavelength tuning range of 37.2 nm. The amplifier similaritons are four-wavelength switchable between 1072.71 nm, 1060.15 nm, 1048.92 nm, and 1037.26 nm, with a total central wavelength tuning range of 35.15 nm. The Vernier effect filter outperforms traditional fiber interference filters in terms of sensitivity, facilitating more convenient wavelength switching and offering a broader tuning range for the ytterbium-doped mode-locked fiber lasers. This laser has a variety of potential applications in high-capacity fiber communication systems, biomedical imaging, and basic science research.

## Figures and Tables

**Table 1:** Comparison of the stretch amount and tuning range of the fiber laser described in this paper with those reported in the literature.

Ref.	Structure	Gain Fiber	Laser Type	Tuning Range	Stretch Amount
[18]	SMF-SCF-SMF	Yb-doped	Mode-locked fiber laser	12.36 nm	0.29 mm
[29]	SMF-MMF-SMF	Yb-doped	Mode-locked fiber laser	12.00 nm	0.504 mm
[30]	SMF	Er-doped	Mode-locked fiber laser	4.40 nm	0.10 mm
Current work	SMF-SCF-SMF	Yb-doped	Mode-locked fiber laser	37.2 nm	0.08 mm

## Data Availability

The original contributions presented in the study are included in the article, further inquiries can be directed to the corresponding author.
